# Pain after Interventional Radiology in Oncology: A Case-Control Study from a 5-Year Cohort

**DOI:** 10.3390/cancers14112576

**Published:** 2022-05-24

**Authors:** Narimane Ayaden, Philippe Sitbon, Arnaud Pages, Lambros Tselikas, Jean-Louis Bourgain

**Affiliations:** 1 Gustave Roussy, Service d’Anesthésie, F-94805 Villejuif, France; narimene.ayaden@gmail.com (N.A.); jean-louis.bourgain@gustaveroussy.fr (J.-L.B.); 2 Gustave Roussy, Département de Biostatistiques, F-94805 Villejuif, France; arnaud.pages@gustaveroussy.fr; 3 Gustave Roussy, Interventional Radiology, F-94805 Villejuif, France; lambros.tselikas@gustaveroussy.fr

**Keywords:** postoperative pain, interventional radiology, postoperative analgesia, cancer pain

## Abstract

**Simple Summary:**

Few reports address post-procedural pain after interventional radiology in oncology. All patients treated from 2015 to 2019 in interventional radiology were included in a retrospective cohort study. In an additional case-control study, patients reporting strong or intolerable pain were compared to those with no pain. From 4411 procedures, severe pain was more frequent in women (6% vs. 4%) and the youngest patients, after general anesthesia, arterial embolization, limb cementoplasty, osteosynthesis, and abdominal tumor ablation. In the case-control study, long-term opioid use, duration >160 min, and high-dose remifentanil were risk factors for a high pain level. Intraoperative ketoprofen was associated with a decreased high pain level incidence. Immediate severe post-procedural pain was a risk factor for severe pain in wards until discharge. Severe post-procedural pain was observed, particularly after specific interventional radiology procedures, and may be prevented by injecting intraoperative analgesics.

**Abstract:**

Background: Interventional radiology plays a major role in oncology both for curative and palliative treatment, but few reports address post-procedural pain. The purpose of this study was to quantify postoperative pain after interventional radiology procedures in oncology and to identify major pain-associated pre and intraoperative factors. Methods: From 2015 to 2019, all patients treated with interventional radiology were included retrospectively in a cohort study. Anesthetic protocols were standardized by the type of radiological procedure. Demographic data, preoperative treatments, analgesic agents, pain score levels, and morphine consumption from the post-anesthesia care unit (PACU) to hospital discharge were collected from databases. In an additional case-control study, patients reporting strong or intolerable pain in PACU were compared to those with no pain. Matching to control cases was based on the type of intervention, sex, and age. Results: From 4411 procedures, severe pain in PACU was more frequent in women (*p* < 0.04) and the youngest patients (*p* < 0.0001), after general anesthesia (*p* < 0.0001). Higher pain levels were associated with certain procedures, such as arterial embolization, limb cementoplasty, osteosynthesis, and abdominal tumor ablation, and when the intervention duration exceeded 160 min (*p* = 0.038). In the cohort study, high-dose remifentanil (≥0.055 µg/kg/min) was a risk factor for post-procedural high pain levels (*p* < 0.001). Intraoperative ketoprofen was associated with a decrease in high pain level incidence (*p* < 0.0001). Severe pain in PACU was a risk factor for severe pain in wards from day 0 until discharge. Conclusion: Severe pain depends on the type and duration of interventional radiology, type of anesthesia, and preoperative use of opiates. Limiting doses of remifentanil and injecting intraoperative analgesics, especially ketoprofen, may reduce the incidence of post-intervention severe pain.

## 1. Introduction

Interventional radiology (IR) is increasingly being offered to high-risk patients as an alternative to surgery [1]. These minimally invasive procedures are thought to induce fewer postoperative complications and reduce the length of hospital stay.

The scope of IR in oncology includes diagnostic (biopsies) or therapeutic procedures, both guided by imaging methods (radio, ultrasound, scanner, magnetic resonance). It can be used for direct destruction of tumors by radiofrequency, cryotherapy, laser, microwave, treatment of complications (e.g., abscess drainage, arterial embolization), adjuvant treatment, such as regional chemotherapy injection, and palliative or symptomatic therapy, such as pain treatment or dilation of stenosis ducts.

Modalities of anesthesia vary by procedure, whether local anesthesia, sedation, general anesthesia (GA), or regional anesthesia (RA). There is no consensus in the literature regarding the use of a particular anesthesia technique for a specific IR procedure, and each team of radiologists and anesthetists must determine its own anesthesia protocol.

Published studies addressing post-procedural pain control and its effect on the oncology patient are uncommon in IR. One study evaluated 150 patients for objective pain scores 24 h before and after nine different interventional procedures, observing significant increases in pain after some procedures [2]. The limited number of patients included in these studies makes it difficult to identify clear risk factors or how to prevent these outcomes [3].

Few studies have focused on determining associated factors for postoperative pain and their prevention by intraoperative injection of pain medication, as for surgery [1]. When such studies have been carried out, they have focused on the postoperative follow-up of a specific treatment, such as chemo-embolization [4], cementoplasty [5], or arterial embolization [6]. The problem of post-IR pain assessment is made even more complex because some procedures are indicated to treat a painful condition.

The purpose of the first part of this work, a cohort study, was to assess retrospectively the incidence of postoperative pain after IR by the type of intervention, demographic characteristics, and anesthesia procedures from a structured database in a cancer center. In a second part, a case-control study, we compared patients reporting the most pain with those reporting none in the post-anesthesia care unit (PACU) regarding pre and intraoperative risk factors and the course of their pain until hospital discharge.

## 2. Materials and Methods

### 2.1. Patients and Procedures

We performed a cross-sectional observational study using medical data from a comprehensive cancer center. All patients who underwent an IR procedure during January 2015 to December 2019 were included. Patients under the age of 18 years and those who had an associated surgical procedure at the same time were excluded. Each patient gave written consent to authorize health care teams to use anonymized data from their medical records for investigation. This current analysis was approved by the French Ethics Committee for Research in Anesthesiology and Intensive Care with IRB approval n° IRB 00010254-2021-094.

Data were analyzed only for patients who had received GA, RA, sedation, or monitored care. Procedures under local anesthesia without anesthetic supervision were not included. The anesthesia technique was standardized and varied according to the IR procedure: simple monitoring, RA, sedation (remifentanil using effect-side target concentration infusion; TCI), and GA. The standard analgesia protocol included intraoperative injection of paracetamol and tramadol in all patients, and nefopam was added in all patients under GA. Ketamine (bolus and/or infusion), ketoprofen, and morphine were injected according to the anesthesiologist’s choice. GA was begun with propofol bolus (2–2.5 mg/kg), and maintenance was carried out by halogenated agents (sevoflurane or desflurane), and rarely by TCI propofol. Atracurium was adjusted according to monitoring. The same analgesic protocol was used for surgical and IR patients and was adapted to the types of intervention.

### 2.2. Design of the Studies

All procedure data for patients aged 18 years and older were included in the cohort study. From the cohort, patients assessing their pain at 3 (strong) or 4 (intolerable) using an analog or subjective scale from 0 to 4 (numerical verbal pain score; NVS) were compared as cases to patients reporting no pain in the PACU (score 0) as controls in a case-control study. Cases and controls were matched by the type of intervention and then by sex and age.

### 2.3. Outcomes

Demographics, age, weight, height, and preoperative analgesic treatments (with a particular focus on analgesic, opioid, psychotropic (antidepressant, antiepileptic), and benzodiazepine treatments) were extracted from the anesthesia consultation.

Intraoperative and PACU medical data were collected on Centricity Anesthesia software (General-Electric-France Buc France), for both IR and surgical patients. This tool has been described in detail previously [7]. Events, pain scores, anesthesia techniques and administered drugs were recorded during the procedure by the anesthesia team in structured fields where the cumulative dose and units are fixed. Age, sex, and type of the intervention entries were mandatory. Based on the team’s experience, the percentage of missing data was lower than 10% and did not affect average values and distribution [8].

The following data were collected from the Centricity software: age, weight, height, American Society of Anesthesiologists class, type of anesthesia, and amounts of morphine and other analgesic agents (paracetamol, nefopam, tramadol, ketoprofen, ketamine) injected during the procedure and in the PACU, as well as the duration of the procedure. The doses of ketamine and remifentanil were transmitted through an interface with an infusion pump.

NVS from 0 to 4 (0, no pain; 1, low pain; 2, medium pain; 3, strong pain; and 4, excruciating pain) was measured in the PACU at least on arrival and exit, and the maximum score was retained for this study [6]. If the score could not be acquired because of consciousness impairment, the value was deemed non-evaluable.

After leaving the PACU, the consumption of morphine and other analgesic agents, pain intensity as measured by a numerical rating scale of 0–10 was collected from the medical data management software DxCare (Medasys Dedalus, Le Plessis Robinson, France) at D0, D1, and hospital discharge. The numerical rating scale was only available on this software and the values from the pain assessment scales were converted to the 0–4 scale to homogenize them with the pain scale used in the PACU and on the wards.

### 2.4. Statistical Analysis

In the cohort study, the categorical variables are reported as numbers and percentages; the continuous variables are expressed as means and standard deviations. For comparisons between different pain groups (score 0 vs. score 1 vs. score 2 vs. scores 3 or 4), we used analysis of variance for continuous variables and the chi^2^ test for categorical variables. The influence of the interventional procedure was analyzed by technique type and target organ. Finally, we analyzed more specifically the relationship between pain level in the PACU and the amount of remifentanil, expressed as µg/kg/min, to identify a possible threshold at which the pain would be increased.

In the case-control study, we performed bivariate then multivariate analyses (logistic regressions) to determine which factors were associated with the main outcome (severe pain, score 3 or 4 vs. no pain, score 0). All of the variables significantly associated with the outcome at a threshold of 0.2 in bivariate analyses were included in the multivariate models. We then used a backward stepwise regression procedure to exclude variables from the model.

Differences were considered statistically significant at *p* < 0.05. All analyses were carried out using SAS version 9.4 software (SAS Institute, Inc., Cary, NC, USA).

## 3. Results

### 3.1. Cohort Study

**Figure 1:**
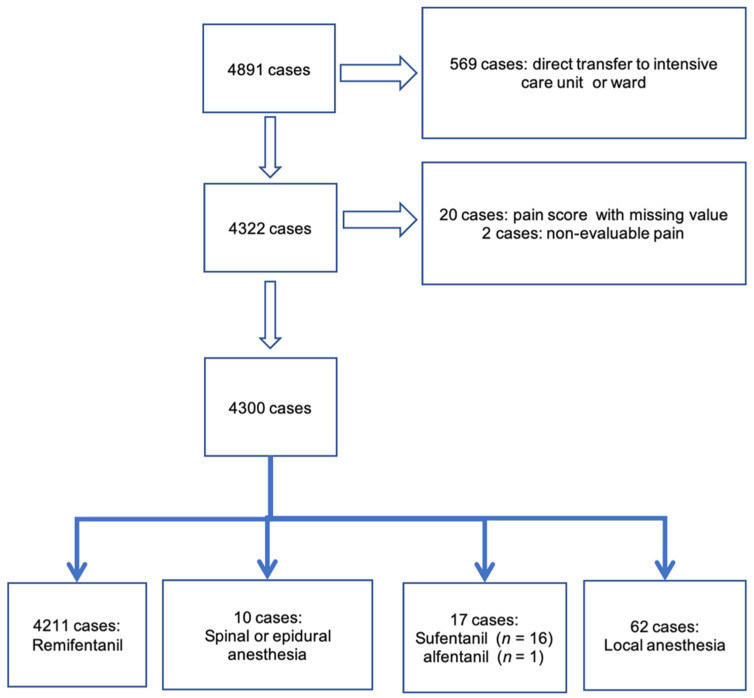
Flow chart of the population treated with interventional radiology and anesthesiology.

For this 5-year period, 4891 IR procedures under anesthesia were extracted. We removed 569 procedures from the study for patients who did not switch to the PACU (a direct transfer to ward or intensive care unit), 20 because no pain score was recorded, and 2 because pain remained non-evaluable during the stay in the PACU (Figure 1). A total of 62 cases involved local anesthesia, or compartment or plexus block. To improve the homogeneity of the population, statistical calculations were made only for patients with remifentanil infusion.

In the cancer patients, the incidence rates of the most important pain (score 3–4) were comparable for IR and surgery (all specialties combined) during the same period (5.0% for IR and 4.9% for surgery), and lower than for abdominal surgery (9.7%). Only 17 patients rated their maximum pain at level 4 (0.4%) and 194 patients at level 3 (4.6%) during their stay in the PACU. To ensure enough patients for analyses, we grouped these patients into the same class “group 3–4.”

The cohort characteristics are described in Table 1. Pain scores 3–4 were statistically more frequent in women (*p* < 0.04; Table 1). The age of patients with pain scores of 3–4 was lower than for those with scores of 0–2 (*p* < 0.0001). NVS was higher after GA than after other types of anesthesia (6.0% vs. 2.7%, *p* < 0.0001). Compared with patients reporting lower pain levels, those with a score of 3–4 in the PACU received significantly less analgesia (except for tramadol) preventively during the procedure.

**Figure 2:**
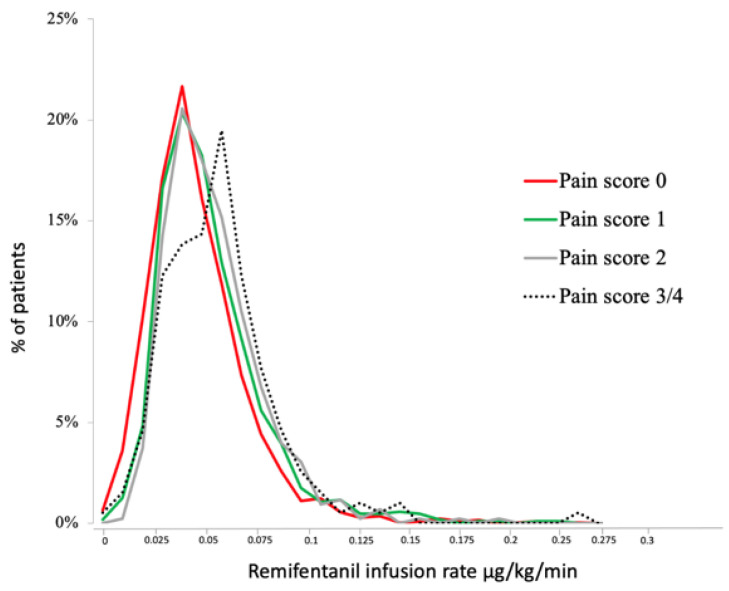
Relationship between the average remifentanil infusion rate during the procedure and maximal pain score recorded in PACU.

The incidence of pain 3–4 was less than 5% for most procedures and exceeded 5% for several types of procedures, however, included arterial embolization, bone treatment (cementoplasty and osteosynthesis), and tumor ablation (Table 1). The postoperative pain score was comparable after tumor ablation regardless of the technique used (i.e., microwave, radiofrequency, or cryotherapy). Postoperative pain was higher when the treatment involved the bones of the limbs (10%) rather than those of the spine and the pelvis (4.5%), and the kidney and liver rather than the lung (*p* < 0.001). Patients with NVS 3 and 4 had a higher average infusion rate of remifentanil. As Figure 2 indicates, a remifentanil threshold of 0.055 µg/kg/min was associated with more severe pain (Table 2). For rates below this threshold, we defined three groups according to terciles (<0.032 µg/kg/min, between 0.032 and 0.043 µg/kg/min, and between 0.043 µg/kg/min and 0.055 µg/kg/min). Above the threshold of a 0.055 µg/kg/min remifentanil infusion rate, the risk for high pain became significant (Table 2).

In PACU, patients with a high pain level received more rescue analgesics than patients reporting no pain. Among patients rating pain 3–4, 90% received morphine in the PACU versus 0.6% of patients with a score of 0. Those with pain at 3–4 also had more nonsteroidal anti-inflammatory drug (NSAID) use at 24% versus 4% for those with a score of 0 and more ketamine use at 25% versus 0.3% with a score of 0.

### 3.2. Case-Control Stud

The 211 patients with NVS 3 (*n* = 194) and 4 (*n* = 17) were matched to 211 patients with NVS 0, according to the type of procedure, sex, and age. The number of patients for whom the indication for IR was justified to treat cancer pain was comparable in the two groups, at 39 in the case group and 28 in the control group (*p* > 0.1).

In the multivariate analysis, long procedure duration, GA, a high remifentanil infusion rate, and a preoperative history of opioid consumption were associated with more severe pain. Conversely, intraoperative use of NSAIDs was associated with less pain (Table 2). A preoperative analgesic or psychotropic medication for pain or psychiatric disorders was not associated with more severe pain.

**Figure 3:**
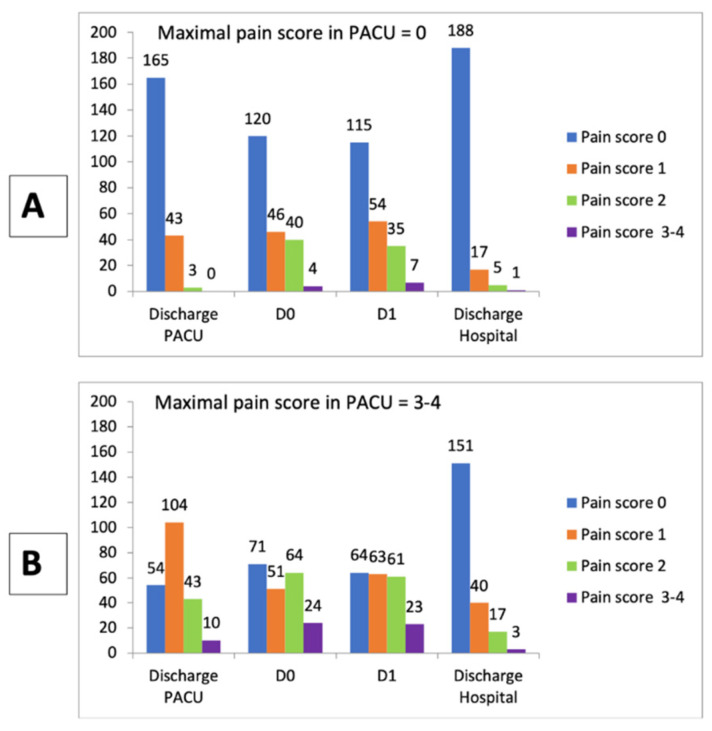
Time course of pain scores from PACU discharge to hospital discharge in case-control study: control (**A**): max pain score in PACU = 0) vs. case (**B**): max pain score in PACU = 3–4). *p*< 0.001 for scores at PACU discharge, D0, D1, and *p* = 0.019 at hospital discharge (Fisher test).

In the case-control study, after PACU discharge until the end of the hospital stay, patients with NVS 3–4 received more morphine than control patients (78 vs. 50) and more NSAIDs (54 vs. 32). The difference was significant for all analgesics (*p* < 0.0001) except for ketamine. Despite the application of this analgesic treatment, pain scores measured at PACU exit and in the wards until hospital discharge was lower (*p* < 0.02) in the control group (NVS 0) than in the case group (NVS 3–4) (Figure 3).

## 4. Discussion

This large database study, with 4211 enrolled patients, is the first to explore postoperative pain after IR for multiple types of cancer procedures. Despite the minimally invasive nature of IR, some patients experienced severe pain in the PACU. As has been widely reported, the incidence of severe pain was higher in women and men than in [9] and related to a younger age [10]. The first part of this work involved comparisons according to different pain scores in the PACU and the relationship of high pain scores (3 or 4) with potential risk factors. To strengthen the methodology, based on the number of patients with high pain scores, we conducted a case-control analysis with logistic regressions to focus on specific risk factors for high pain and follow-up until hospital discharge.

The incidence of severe pain was not higher in patients for whom the radiological procedure was indicated to treat pain or to treat cancer. The likely reason is the delayed analgesic efficacy of these treatments [11].

We found greater pain in patients with preoperative opiates, as already seen for surgical patients, possibly because of opioid-induced hyperalgesia (OIH) [12]. This result could suggest a need for more systematic preventive use of ketamine. Neurotropic drugs were not a risk factor for severe pain after IR, in contrast with previously reported findings with all surgical and IR patients in our hospital [13]. In this cohort, the small number of patients with these treatments could underlie a lack of power to identify this risk factor.

The type of procedure performed influenced the intensity of postoperative pain. Based on an arbitrarily set incidence threshold of 5%, cementoplasties, osteosynthesis, tumor ablations, and arterial embolizations led to more intense postoperative pain.

The incidence of post-IR pain in cancer patients has not been studied in large cohorts. However, some studies have evaluated pain treatment after cementoplasties [5], embolization [6], and tumor ablation [14] and concluded that pain control was inadequate and that pain medications should be prescribed more liberally during and after tumor ablation [3] or percutaneous osteosynthesis [15]. As previously reported, the type of techniques used to treat tumors did not play a predominant role, and pain level and opiate consumption are comparable after radiofrequency and electroporation [16]. In our study, a high pain level was observed after certain procedures, especially on the limbs, which might be better controlled by RA, calling for further studies.

According to our protocols, almost all patients on GA or sedation received remifentanil. Remifentanil, at high doses, is deemed to increase postoperative pain by a dose-dependent OIH mechanism [9]. This hyperalgesic effect has been reported after surgery [17], however, the duration [18] and the dose [19] of remifentanil-induced hyperalgesia remains controversial. Our findings suggest a relationship between the highest doses of remifentanil and the worst pain in the PACU. Both the amount and duration of remifentanil infusion played a significant role in the univariate analysis. In practice, these results suggest limiting remifentanil doses and using multimodal analgesia.

In both the cohort and case-control studies, intraoperative injection of ketoprofen significantly decreased the incidence of major pain. NSAIDs use is highly recommended for the prevention of postoperative pain [20]. Perioperative administration of parecoxib has proved effective in reducing pain (level of pain and morphine consumption) after chemo-embolization of hepatocellular carcinoma [21] and in vertebroplasty for spine metastasis [22]. The synergistic action of NSAIDs with opioids encourages the use of this drug class more systematically [13]. In the absence of contraindication, it makes sense to administer prophylactic NSAIDs more systematically for IR procedures, such as embolization, tumor ablation, or treatment of bone lesions.

In the cohort study, patients with a score of 3–4 received more intraoperative morphine than patients reporting no pain. This pattern was not confirmed in the case-control study, where this factor became insignificant. The discrepancy may be explained by the more frequent preoperative opioid use in the case group, as these patients usually need more intraoperative morphine to control postoperative pain, as recommended during surgery [23].

No definitive conclusion could be established for the efficacy of other intraoperative analgesic drugs because of the discrepancy between the statistical results of the cohort and case-control studies. The small number of patients in the case-control study may have reduced statistical power in comparison with the cohort study.

Our clinical protocol in the PACU recommended using analgesic agents that were not administered during the procedure. Postoperative administration of ketamine, morphine, and ketoprofen in the PACU helped to control pain and very few patients (three with NVS 2 and none with a score of 3–4) were experiencing pain when discharged from the PACU. A total of 90% of patients with an NVS score of 3–4 received morphine in the PACU versus 0.6% of patients with a score of 0.

As shown in Figure 3, patients with a score of 3–4 in the PACU presented a higher pain score than control patients (score of 0 in PACU) until hospital discharge. Even among surgical patients, those with pain in the PACU have a higher risk of pain at D1 or beyond with the risk of chronic pain [24]. Increased pain on D1 is also observed after surgery and justifies a specific follow-up of these at-risk patients in the hospital ward [25].

This retrospective study involved many patients whose management followed a pre-established protocol. The small amount of missing data attests to good patient follow-up. The case-control analysis mitigates the negative aspects of this type of retrospective study by weighting several variables, such as sex or intervention. The advantage of a retrospective study using a structured database including many interventions is to reflect day-to-day practice and highlight outlier patients, such as those with excruciating pain in the PACU (17 patients with NVS 4 during the 5-year period).

## 5. Conclusions

This study focused on postoperative pain after IR in a cancer treatment context. Pain was more significant after certain types of procedures, including tumor ablation, treatment of limb bone lesions, and arterial embolization. The incidence of severe pain after IR was close to 5% and increased in patients with preoperative opiates. High remifentanil doses were associated with a higher risk of developing severe pain in the PACU. Intraoperative administration of analgesics, particularly ketoprofen, reduced this incidence. Beyond the PACU until hospital discharge, patients with scores of 3–4 remained more painful than others despite the painkillers’ administration. This pattern encourages the adoption of a preventive approach, such as the limitation of remifentanil doses, more systematic use of NSAIDs and RA, and improved postoperative follow-up in the wards for patients with a high level of pain in the PACU.

## Figures and Tables

**Table 1:** Pain score according to general population characteristics, type of anesthesia, type of procedure.

Variable			Number of Patients	Number (%) of Patients/Pain Score				*p*-Value
				0	1	2	3–4	
Demographic Caracteristics	Sex	Female, n (%)	2117	1260 (59.5)	523 (24.7)	208 (9.8)	126 (6.0)	0.04
		Male, n (%)	2094	1274 (60.8)	515 (24.6)	220 (10.5)	85 (4.1)	
	Age	Years, mean (SD)	4211	60 (14)	60 (15)	59 (14)	55 (14)	0.0001
	Duration	Minutes, mean (SD)	4211	131 (53)	143 (51)	143 (50)	155 (62)	0.0001
	Weight	kg, mean (SD)	4211	71 (16)	71 (17)	69 (15)	69 (15)	0.07
	Height	cm, mean (SD)	4211	170 (9)	169 (9)	168 (9)	168 (9)	0.04
Type of Anesthesia, n (%)	General		2966	1658 (55.9)	801 (7.0)	328 (11.1)	179 (6.0)	0.0001
	Sedation		1203	850 (70.7)	225 (18.7)	97 (8.1)	31 (2.6)	GA versus other types of anesthesia
	Regional and local anesthesia *		41	22 (53.7)	12 (29.3)	2 (4.9)	1 (2.4)	
Intraoperative Analgesic	Paracetamol	n (%)	3942 (100)	2363 (93.3)	979 (94.3)	409 (95.5)	191 (91)	0.0399
		Dose in mg, mean (SD)	0.99 (0.06)	0.99 (0.05)	0.99 (0.06)	0.99 (0.06)	0.99 (0.06)	
	Tramadol	n (%)	3197 (100)	1923 (75.9)	817 (78.7)	321 (75)	136 (65)	0.2688
		Dose in mg, mean (SD)	95 (15)	95 (15)	95 (15)	94 (17)	96 (14)	
	Ketoprofen	n (%)	1914 (100)	1098 (43.3)	501 (48.3)	241 (56.3)	74 (35)	0.0001
		Dose in mg, mean (SD)	95 (15)	95 (15)	96 (14)	94 (17)	97 (11)	
	Ketamine	n (%)	2009 (100)	1134 (44.7)	545 (52.5)	245 (57.24)	85 (41)	0.0001
		Dose in mg, mean (SD)	20 (10)	19 (7)	20 (9)	18 (6)	20 (7)	
	Nefopam	n (%)	3478 (100)	2054 (81)	912 (87.9)	353 (82.5)	159 (75)	0.0001
		Dose in mg, mean (SD)	20 (1)	20 (2)	20 (1)	20 (0)	20 (0)	
	Morphine	n (%)	2171 (100)	1214 (48)	588 (57)	247 (58)	122 (58)	0.0001
		Dose in mg, mean (SD)	5.1 (2.4)	4.8 (2.1)	4.9 (2.2)	5.5 (2.9)	5.8 (2.8)	
Procedure by Type	Embolization	Chemo-embolization	307	202 (65.8)	60 (19.5)	36 (11.7)	9 (2.9)	N.A.
		Arterial embolization	246	149 (60.6)	51 (20.7)	29 (11.8)	17 (6.9)	N.A.
	Bone treatment	Cementoplasty total	1036	622 (60.0)	260 (25.1)	98 (9.5)	56 (5.4)	N.A.
		Osteosynthesis	148	68 (45.9)	38 (25.7)	28 (18.9)	14 (9.5)	N.A.
	Tumor ablation	Cryotherapy	709	382 53.9)	202 28.5)	82 (11.6)	43 (6.1)	N.A.
		Microwave	88	41 (46.6)	31 (35.2)	12 (13.6)	4 (4.5)	N.A.
		Radiofrequency	647	353 (54.6)	182 (28.1)	78 (12.1)	34 (5.3)	N.A.
	Biopsy		123	85 (69.1)	27 (22.0)	6 (4.9)	5 (4.1)	N.A.
	Gastrostomy, nephrostomy		190	137 (72.1)	36 (18.9)	15 (7.9)	2 (1.1)	N.A.
	Biliary prosthesis or drainage		300	177 (59.0	82 (27.3)	27 (9.0)	14 (4.7)	N.A.
	Vascular **		310	236 (76.1)	46 (14.8)	15 (4.8)	13 (4.2)	N.A.
	Others ***		107	82 (76.6)	23 (21.5)	2 (1.9)	0.0	N.A.

* Others: endoscopy, electroporation, ductal dilation, urinary derivation, vascular prosthesis, infiltration, alcohol infiltration, prosthetic change, fibrinolysis, thrombolysis, catheter repositioning. ** Intra-arterial chemotherapy; radioembolization; cava filter; arteriography. *** Endoscopy, electroporation, ductal deletion, urinary derivation, vascular prosthesis, infiltration, alcohol infiltration, prosthetic change, fibrinolysis, thrombolysis, catheter repositioning.

**Table 2:** Case and Control Population Characteristics.

Parameters	Crude Odds Ratio [95% CI]	*p*-Value	Adjusted * Odds Ratio [95% CI]	*p*-Value
**Duration of the procedure (minutes)**				
First tercile (duration < 118 min)	Ref.	0.016	Ref.	0.038
Second tercile (118 min ≤ duration < 160 min)	1.01 [0.63–1.61]		0.94 [0.57–1.54]	
Third tercile (duration ≥ 160 min)	1.83 [1.14–2.94]		1.70 [1.03–2.83]	
**Weight (kg)**				
First tercile (weight < 62 kg)	Ref.	0.919	-	-
Second tercile (62 kg ≤ weight < 75 kg)	1.00 [0.63–1.60]		-	
Third tercile weight ≥ 75 kg)	0.92 [0.58–1.47]		-	
**Height (m)**				
First tercile (height < 1.64 m)	Ref.	0.709	-	-
Second tercile (1.64 m ≤ height < 1.75 m)	0.84 [0.52–1.35]		-	
Third tercile (height ≥ 1.75 m)	0.85 [0.53–1.34]		-	
**Body mass index (BMI, kg/m²)**				
Underweight (BMI < 18.5)	Ref.	0.723	-	-
Normal (18.5 ≤ BMI < 25)	0.92 [0.46–1.86]		-	
Overweight (25 ≤ BMI < 30)	1.21 [0.57–2.58]		-	
Obese (BMI ≥ 30)	0.97 [0.43–2.21]		-	
**ASA Physical Status Classification System**				
ASA I or ASA III	Ref.	0.601	-	-
ASA III or ASA IV or ASA V	1.12 [0.74–1.68]		-	
**Anesthesia**				
No general anesthesia	Ref.	0.016	Ref.	0.022
General anesthesia	1.83 [1.12–2.98]		1.84 [1.09–3.10]	
**Remifentanil dose (µg/kg/min)**				
Low dose (dose < 0.032 µg/kg/min)	Ref.	0.019	Ref.	0.005
Medium dose (0.032 µg/kg/min ≤ dose < 0.043 µg/kg/min)	0.98 [0.54–1.80]		1.16 [0.62–2.20]	
High dose (0.043 µg/kg/min ≤ dose < 0.055 µg/kg/min)	1.73 [0.92–3.24]		1.99 [1.03–3.83]	
Very high dose (dose ≥ 0.055 µg/kg/min)	1.90 [1.18–3.08]		2.33 [1.39–3.88]	
**Intraoperative medication (yes/no)**				
Paracetamol	0.53 [0.25–1.13]	0.098	-	-
Nefopam	0.65 [0.41–1.04]	0.075	-	-
Tramadol	1.02 [0.69–1.52]	0.919	-	-
Ketoprofen (NSAID)	0.53 [0.36–0.78]	0.001	0.48 [0.32–0.73]	0.001
Morphine	1.34 [0.91–1.96]	0.142	-	-
Ketamine	0.86 [0.58–1.26]	0.428	-	-
**Intraoperative morphine dose (mg)**	1.07 [1.01–1.14]	0.023	-	-
**Medication taken before the procedure (yes/no)**				
Long-term analgesic use	1.48 [0.87–2.51]	0.144	-	-
Long-term use of psychotropic drugs	1.03 [0.66–1.59]	0.911	-	-
Long-term opioid use	1.60 [1.06–2.44]	0.027	1.56 [1.00–2.44]	0.050
Use of benzodiazepines before the procedure	1.41 [0.79–2.51]	0.245	-	-
**History of anxiety or psychiatric illness**	0.48 [0.22–1.04]	0.063	-	-

* From the final reduced model (logistic regression) after variable selection with backward stepwise procedure. Legend: Ref., reference; [95CI, 95%] confidence interval; BMI, body mass index; ASA, American Society of Anesthesiologists; NSAID, non-steroidal anti-Inflammatory drug.

## Data Availability

The data presented in this study are available on request from the corresponding author. The data are not publicly available due to medical confidentiality.
